# The relationship between peer relations, self‐rated health and smoking behaviour in secondary vocational schools

**DOI:** 10.1002/nop2.260

**Published:** 2019-03-23

**Authors:** Hanna Aho, Anna‐Maija Koivisto, Eija Paavilainen, Katja Joronen

**Affiliations:** ^1^ Faculty of Social Science, Health Sciences University of Tampere Tampere Finland; ^2^ Department of Musclosceletal Diseases Tampere University Hospital Tampere Finland; ^3^ Tampere University of Applied Sciences Tampere Finland

**Keywords:** adolescent health, bullying, health promotion, inequalities of health, school health, school nursing, smoking

## Abstract

**Aims:**

To examine the association between peer relations, self‐rated health and smoking behaviour in vocational school setting.

**Background:**

Smoking in adolescence causes health and socioeconomic inequality in adulthood. There is evidence that smokers are physically less active, have lower academic aspirations and perceive poorer health than non‐smokers.

**Method:**

The study was conducted in spring 2013 and involved 34,776 vocational students who took part in the School Health Promotion Study in Finland. The associations between adolescent smoking habits and peer relations and smokers' self‐rated health were studied adjusting for the respondents' age, parental education and family type.

**Results:**

A substantial proportion of the respondents, 37% of the girls and 36% of the boys, reported smoking daily, 15% of the girls and 14% boys smoked occasionally with a further 15% of the girls and 13% of the boys stating that they were ex‐smokers. Of the girls, 33% and 38% of the boys were non‐smokers. Adjusted multinomial regression revealed that having a close friend or friends predicted smoking among girls and boys. Additionally, the adjusted model indicated that being a bully and/or a bully + bully‐victim was associated with smoking behaviour in boys only. Boys and girls who rated their health as moderate or poor were more often daily smokers; in girls, this was also the case in occasional smokers.

**Conclusion:**

Smoking prevention aimed at vocational schools should take into consideration the norms and expectations related to peer relations which strongly influence adolescents' smoking habits.

**Why is this research needed?**

- Tobacco smoking is a preventable cause on health inequality and premature death. Youths studying for blue‐collar trades in vocational schools smoke significantly more than those youths who have selected academically orientated upper secondary school, that is high school.
- There are very few good quality studies conducted on smoking among vocational students.

**What are the key findings?**

- Tobacco smoking is disproportionately prevalent among vocational students.
- Friendships are related to smoking among vocational school students.
- Bullies and/or bullies who are also bullying victims are more frequently smokers than students who do not participate in bullying behaviour among boys only.
- Adolescents that rate their health as moderate or bad are more likely smokers.
- Relationship between peer relations and adolescents' smoking is complex also in vocational schools; further research with multiple methods will be needed to clarify this association.

**How should the findings be used to influence policy/practice/research/education?**

- Enhancing school activity in school premises aiming social bonding to all schoolmates and connectedness to school might have a great impact of creating healthy study environment.
- Both community and school nurses are well positioned to provide education and support at reducing smoking and to promote methods for effective smoking cessation.
- Strategies to reduce socioeconomic inequalities in smoking should involve aspects of peer relationships.

**Impact statement**

Smoking in adolescence leads to health inequality in adulthood. According to this study, health inequality is evident much earlier, already in adolescence. School health nurses and community health nurses have an unique opportunity to promote effectively healthy study environments by taking account of peer relations at school.

## 1 INTRODUCTION

Smoking among adolescents is a major public health concern as smoking poses many health risks such as substance use (O'Loughlin, Dugas, O'Loughlin, Karp, & Sylvestre, 2014) and lower level of physical activity (Kauranen, 2013) leading to health inequalities in adulthood (World Health Organization, 2015). Furthermore, adolescents who smoke have been associated with negative behaviours, such as truancy (Vaughn, Maynard, Salas‐Wright, Perron, & Abdon, 2013) and bullying (Luk, Wang, & Simons‐Morton, 2012). More than four out of every five U.S adult smokers have begun smoking before 18 years of age and smokers who start smoking at a young age are more likely to smoke as adults (American Lung Association, 2016). High school students who are acquainted with peers and family members who are smokers have been found to report more positive symptoms from their initial smoking experience (Okoli, Richardson, & Johnson, 2008). Therefore, although prevention of intergenerational transmission is important, also peer relationships and social relationships should be accounted for while planning research into ways to curb adolescent smoking.

The relationships between peer relations to adolescent smoking are complex. Peer relationships at school and perceptions of belongingness can mitigate the effects of risk factors linked to substance use. Friendships have been found to be protective against substance use, as well as mediating the relationship between social self‐control (Forster, Grigsby, Bunyan, Unger, & Valente, 2015; Tang & Loke, 2012). Thus, social bonding with friends and classmates is highly recommendable, and it is peer selection and influence that have found to precede adolescent and young adult smoking (Jones et al., 2013; Seo & Huang, 2012). It has been demonstrated that adolescents with friends who smoke are likely to smoke themselves or to take up smoking over time (Simons‐Morton & Farhat, 2010).

In Finland, after 9‐year compulsory elementary school, there are two separate types of secondary schools. After the compulsory schooling, 55% of school‐leavers of Finland choose to continue into the academically oriented upper secondary school, which prepares students for graduate education. About 39% will choose vocational schooling and training that is aiming to improve the skills of the workforce and prepare students for specific vocations. The largest fields are technology and transport, business and administration and health and social services leading to professions such as car mechanic, carpenter, sales personnel, practical nurse, that is care assistant, hairdresser or dental laboratory assistant. All study programmes in vocational qualifications take three years (120 credits) to complete. In the initial vocational upper secondary level, there are 52 vocational qualifications, and in all programmes, there is a compulsory minimum of six‐month period of on‐the‐job learning. (Ministry of Education & Culture, Finland, 2016).

According to Belgian research, students in vocational schools have a significant lower sense of belonging than students in academic high school (Van Houtte & Van Maele, 2012). Studying requires more independence on behalf of the student; he/she is responsible for how well or badly they make progress on the road to becoming a skilled professional. Smoking rates among adolescents' studying for different vocations are much higher than among high school students. It was estimated in 2013 that about 36% of those Finnish adolescents learning a specific trade in vocational schools are smoking daily, compared with only 8% of their previous classmates that continued to the academically focused upper secondary school after ninth grade. This relationship has been noted also in other Western countries (Huisman, Werfhorst, & Monshouwer, 2012; Ingholt et al., 2015; Lee, Goldstein, Klein, Ranney, & Carver, 2012; Loukas, Murphy, & Gottlieb, 2008). Furthermore, academic achievement and smoking behaviour exhibit an association, that is individuals with lower levels of academic achievement seem to be more likely to smoke cigarettes (Andersen et al., 2015). A study conducted among vocational school in Finland found some students believing that smoking enhances their social standing and projects an image of a skilled professional (Kauranen, 2013). Blue‐collar workers also smoke significantly more than their white‐collar counterparts. Since vocational training has periods of on‐the‐job learning, the students may mimic the behaviour of their workmates/instructors (Bonevski, Paul, Walsh, Bryant, & Lecathelinais, 2011). Tutors are considered as authorities whose views and example are generally not opposed (Kiri & Catherine, 2018).

### 1.1 Background

Smoking is a multifaceted behaviour influenced by several factors, and undoubtedly, the school environment exerts a critical influence on adolescent well‐being. The theory of triadic influence (TTI) (Flay & Petraitis, 1994; Flay, Petraitis, & Hu, 1999) suggests that adolescents' smoking behaviour is influenced by intrapersonal factors and contextual features but also by socio‐environmental aspects such as friends and family; learning, bonding and normative beliefs. Peer relations and social belonging have a major influence on the adolescent's school perception, that is the so‐called school connection; these factors have been shown to affect educational ambitions, for example decreasing truancy and dropout rates (Crosnoe & Johnson, 2011; Seo & Huang, 2012).

#### 1.1.1 Peer relations

Classmates have been shown to be important to allow vocational students to become engaged with their school (Elffers, Oort, & Karsten, 2012) A lack of peer relations has been shown to result in school dropout (Havik, Bru, & Ertesvåg, 2015). Bullying behaviour exerts a significant detrimental impact on adolescent well‐being; for instance, it is responsible for truancy and dropout, even suicide (Havik et al., 2015; Kelly et al., 2015). Previously, bullying has been shown to be more prevalent among middle school adolescents than older students but it does persist also in the final school years (Azagba, 2016; Radliff, Wheaton, Robinson, & Morris, 2012). There is research evidence indicating that bullying is related to smoking behaviour (Azagba, 2016; Klein, Cornell, & Konold, 2012; Luk et al., 2012; Niemelä et al., 2011; Radliff et al., 2012). Studies conducted in Australia and the USA among middle and high school students found that both bullies and bullies that have been bullied themselves reported the greatest levels of substance misuse and smoking while bullying victims and students not involved in bullying were less likely to abuse substances (Kelly et al., 2015; Radliff et al., 2012) Furthermore, those bullied during childhood were more likely to be regular smokers by the age of 18 (Niemelä et al., 2011).

#### 1.1.2 Self‐rated perceived health

Adolescent students' well‐being is related to their subjective social status (Zorotovich, Johnson, & Linn, 2016), but the social status gained by smoking does not seem to correlate with perceived or self‐rated health (Hansen, Lindström, & Rosvall, 2015). Daily and occasional smokers have reported more physical and psychological complaints and lower quality of life than never smokers (Dube, Thompson, Homa, & Zack, 2013; Hansen et al., 2015; Wang, Ho, Lo, Lai, & Lam, 2012). Previously, early smoking initiators have reported poorer health than later initiators and this poorer self‐rated health remains even after smoking cessation among boys who started to smoke at an early age (Hansen et al., 2015).

## 2 AIMS

There are studies conducted in primary and secondary schools examining the association between peer relations with smoking but fewer studies have investigated peer relations related to adolescent smoking in the vocational school setting, even though there has been a traditionally high prevalence of vocational school students who are smokers. Furthermore, smokers' self‐rated health has not previously been studied in this setting. In this study, we will examine whether: (a) peer relations; and (b) self‐rated health are associated with adolescents' daily, occasional and former smoking behaviour in a vocational school setting.

## 3 DESIGN

This was a secondary data analysis using the data of School Health Promotion Study carried by Institution of National Health and Welfare in Finland. The data were analysed with multinomial regression, cross‐sectional design.

### 3.1 Participants

The target group for this study consisted of 1st (57%) and 2nd (43%) grade students in vocational schools in Finland in 2013. A total of 34,776 students from all 419 vocational schools in Finland completed the questionnaire. The response rate of biennial study was not able to count reliably as the number of students was not inquired from the institutes but from statistics that could only give the total number of adolescents studying in vocational schools. However, this study was not conducted for students in their third year. Furthermore, vocational training is based on long practical training periods and that was not considered when conducting the SHP study. Respondents that were out of school the day of the study were not contacted afterwards. However, in this secondary analysis, the rate of missing values was quite low (between 0.3%–2.3%), with one exception: missing values for parents' education were somewhat higher (mothers' education 3.6% and fathers' education 4.7%) and question whether been bullied 12.5%. Vocational training can be started after the ninth grade of elementary school, but it is also possible to start later. For this reason, age distribution within the 1st and 2nd grades may vary. The respondents were aged between 14–20 (Mean = 17.6, *SD* 0.90). Over half (55.6%) were males (*N* = 19,336) and 44.4% females (*N* = 15,440). To account for potential gender differences, separate analyses were conducted for boys and girls. Sample statistics of selected variables are shown in Table 1.

**Table 1:** Sample statistics of selected variables

Variables	Girls		Boys		*p*
	*N*	%	*N*	%	
Current smoking habit					
Daily	5,613	37.2	6,522	35.6	<0.001
Weekly or less than weekly	2,249	14.9	2,311	12.6	
I have quit smoking	2,254	15.0	2,543	13.9	
Non‐smoking	4,955	32.9	6,948	37.9	
Are you experiencing difficulties in getting along with schoolmates					
Not at all	10,795	56.4	9,370	61.1	<0.001
Rather little	5,978	31.2	4,431	28.9	
Rather much	1,753	9.2	1,082	7.1	
Very much	629	3.3	451	2.9	
At the moment, do you have a close friend with whom you can talk confidentially about almost everything concerning yourself?					
I do not have any close friends	736	4.8	1,844	9.8	<0.001
I have one close friend	3,203	21.0	3,797	20.2	
I have two close friends	4,183	27.4	3,755	19.9	
I have several close friends	7,166	46.9	9,441	50.1	
How often have you been bullied at school during this semester?					
Several times a week	209	1.4	557	2.9	<0.001
About once a week	273	1.8	525	2.7	
Rarely	2,170	14.1	3,159	16.4	
Not at all	12,757	82.8	15,035	78.0	
How often have you participated in bullying other pupils during this semester?					
Several times a week	87	0.6	478	2.5	<0.001
About once a week	148	1.0	524	2.7	
Rarely	1,876	12.2	4,112	21.4	
Not at all	13,295	86.3	14,144	73.4	
Bullying indicator					
Bullied bully	80	0.5	492	2.6	<0.001
Bully	154	1.0	510	2.7	
Victim	401	2.6	582	3.0	
Not bullied not bully	14,746	95.9	17,644	91.8	
Self‐rated health					
Moderate or poor	4,064	26.5	3,251	17.0	<0.001
Fairly good or good	11,286	73.5	15,837	83.0	
Respondents age					
14	26	0.2	13	0.1	<0.001
15−16	3,674	23.7	5,217	27.1	
17−18	9,750	63.4	12,747	66.3	
Family type					
Intact	6,847	45.0	10,359	55.2	<0.001
Co‐parenting/dual residence	662	4.3	1,491	7.9	
Single parent	2,364	15.5	3,127	16.7	
Step family	1,437	9.4	1,712	9.1	
Other type	3,914	25.7	2,080	11.1	
Mother's education level					
Comprehensive school or primary school or no education	2,321	15.4	2,655	14.4	<0.001
Upper secondary school or vocational education	6,550	43.5	7,617	41.2	
Occupational studies in addition to upper secondary school or vocational education	3,166	21.0	3,917	21.2	
University, university of applied sciences of other higher education	3,028	20.1	4,279	23.2	
Father's education level					
Comprehensive school or primary school or no education	3,419	23.0	3,761	20.6	<0.001
Upper secondary school or vocational education	6,953	46.8	8,151	44.6	
Occupational studies in addition to upper secondary school or vocational education	2,248	15.1	2,933	16.0	
University, university of applied sciences of other higher education	2,243	15.1	3,450	18.9	

### 3.2 Data collection

Data from the School Health Promotion Study (SHP) conducted by the National Institute for Health and Welfare in Finland were used in this study. SHP is a nationwide survey of adolescents' health and well‐being and is conducted every other year in March–April. The target group for this study consisted of 1st and 2nd grade students in vocational schools in Finland in 2013. A total of 34,776 students from 419 vocational schools anonymously and voluntarily completed a classroom‐administered questionnaire of comprehensive measures of their health and well‐being under their teacher's supervision. The questionnaire can be found online at http://www.thl.fi/fi/web/thlfi-en/research-and-expertwork/population-studies/school-healthpromotion-study

### 3.3 Ethical considerations

The study was approved by the Institute for Health and Welfare Institutional Review Board in Finland. All students were given a detailed explanation of the study by the research team, and voluntary participation to the study was considered as the informed consent according to local regulations. Respondents anonymously completed on their own a classroom‐administered questionnaire under their teacher's supervision, which most likely added the response rate of the study. Participants were informed of their right to withdraw at any phase of the study.

### 3.4 Measures

*Adolescent smoking* behaviour was originally assessed by two questions: (a) How many cigarettes, pipefuls and cigars have you smoked altogether (none, only one, about 2–50 and over 50)? (b) Which of the following alternatives best describes your current smoking habits? (I smoke once a day or more often, I smoke once a week or more often, but not every day, I smoke less often than once a week, I have quit smoking, I have smoked total of only one time and I have never smoked). These adolescent smoking variables were combined into one variable with response categories: daily smokers (I smoke once a day or more often), occasional smokers (I smoke once a week or less often), those who had quit smoking (I have quit smoking) and non‐smokers (I have smoked a total of only one time or never smoked). A total of 846 respondents had inconsistent responses such as they claimed to be non‐smokers in their response to the first question but claimed to smoke on a daily basis in the second question. All those 846 respondents were excluded from the analysis.

*Peer relations* were measured by two questions. First respondents were asked if they are experiencing difficulties in getting along with their schoolmates with 4‐point scale response categories varying from (1) *not at all* to (4) *very much*. This scale was dichotomized into not at all/rather little and very much/rather much. Secondly, respondents were asked if a student had a close friend with whom the respondent could talk confidentially about almost everything concerning her/him. Response categories were as follows: “I do not have any close friends”; “I have one close friend”; “I have two close friends”; and “I have several close friends.” This measure was dichotomized as “having at least one close friend” and “not having any close friends.” Next, the respondent was asked of how often they had been bullied at school during this school semester. The response category was as follows: “several times a week”; “about once a week”; “rarely”; and “not at all.” Students who responded that they had been bullied weekly (several times a week/about once a week) were considered as being bullied at school and the rest of respondents as not bullied at school. Last question that measured students' peer relations was how often you have participated in bullying other pupils during this semester with response categories:“several times a week”; “about once a week”; “rarely”; and “not at all.” Respondents that bullied other pupils on a weekly basis were considered as bullies.

The association between bullying with adolescents' smoking was explored by clarifying the bullying status. Measurements of being bullied and being a bully were combined to create a *bullying status* to clarify the complex nature of bullying with a new measurement: (a) bullied bull;, (b) bully not bullied; (c) bullied not bully; and (d) not bullied not bully.

In the *Self‐rated perceived health,*the respondent evaluated her/his health. Responses were “good”; “rather good”; “moderate”; and “poor.” Measurements were dichotomized as self‐reported health as being “good/rather good” and” moderate/poor.”.

### 3.5 Data analysis

Cross‐tabulation and chi‐squared tests were performed for categorical variables to establish the proportion of students who smoked daily, those who smoked occasionally, those who had quit smoking and finally those students who were non‐smokers on various peer relations factors as well as with the adolescents' perceived health (Table 2). Adjusted (i.e. multivariate analysis) (Table 3) multinomial logistic regression analyses were then performed to examine and evaluate the associations between smoking and peer relations and perceived health factors. Adolescent smoking was set as a dependent variable and peer relations factors and perceived health factor as independent variables. Adolescents' age, mothers' and fathers' education and family type were set as covariates (Aho, Koivisto, Paavilainen, & Joronen, 2017; Wellman et al., 2016). Daily smokers, occasional smokers and those who had quit smoking were compared with non‐smokers, who were used as the reference group. To account for potential gender differences, separate analyses were conducted for girls and boys.

**Table 2:** Cross‐tabulation

	Girls' smoking									Boys' smoking								
	Smokes daily		Occasionally		Has quit smoking		Non‐smoker		*p*	Smokes daily		occasionally		Has quit smoking		Non‐smoker		*p*
	*N*	%	*N*	%	*N*	%	*N*	%		*N*	%	*N*	%	*N*	%	*N*	%	
Difficulties with mates																		
Rather little or not at all	5,028	37.3	2,017	15.0	2,027	15.0	4,419	32.8	0.140	5,656	35.3	2,035	12.7	2,237	14.0	6,088	38.0	0.879
Rather or very much	540	36.6	218	14.8	219	14.8	499	33.8		808	37.8	254	11.9	282	13.2	794	37.1	
Having a close friend																		
I have a friend or friends	5,362	37.7	2,143	15.1	2,118	14.9	4,611	32.4	<0.001	5,862	36.1	2,115	13.0	2,278	14.0	5,964	36.8	<0.001
Don't have any friends	192	27.4	78	11.1	123	17.5	308	43.9		464	27.5	154	9.1	212	12.6	857	50.8	
Bullied at school																		
Several times a week	92	47.7	15	7.8	33	17.1	53	27.5	0.003	226	46.8	52	10.8	58	12.0	147	30.4	<0.001
Weekly	110	41.8	32	12.2	31	11.8	90	34.2		164	37.5	53	12.1	56	12.8	164	37.5	
Rarely	797	37.6	334	15.7	337	15.9	654	30.8		1,079	35.8	389	12.9	397	13.2	1,147	38.1	
Not at all	4,608	37.0	1,860	14.9	1,849	14.8	4,150	33.3		5,039	35.1	1,806	12.6	2,027	14.1	5,474	38.2	
Participated in bullying																		
Several times a week	43	56.6	8	10.5	9	11.8	16	21.1	<0.001	218	52.9	48	11.7	52	12.6	94	22.8	<0.001
Weekly	70	50.4	22	15.8	19	13.7	28	20.1		187	43.7	63	14.7	55	12.9	123	28.7	
Rarely	876	47.6	311	16.9	263	14.3	392	21.3		1,708	43.6	561	14.3	557	14.2	1,088	27.8	
Not at all	4,614	35.5	1,903	14.7	1,955	15.1	4,512	34.8		4,382	32.4	1,630	12.1	1,872	13.9	5,625	41.6	
Bullying status																		
Bullied bully	41	63.1	4	6.2	6	9.2	14	21.5	<0.001	185	47.9	49	12.7	46	11.9	106	27.5	<0.001
Bully	72	48.3	26	17.4	21	14.1	30	20.1		220	48.5	62	13.7	61	13.4	111	24.4	
Victim	161	41.2	43	11.0	58	14.8	129	33.0		203	38.4	55	10.4	68	12.9	203	38.4	
Not bullied not bully	5,324	36.9	2,165	15.0	2,157	15.0	4,769	33.1		5,881	34.9	2,130	12.6	2,357	14.0	6,499	38.5	
Self‐rated health																		
Moderate or bad	1,914	48.0	555	13.9	503	12.6	1,018	25.5	<0.001	1,383	44.7	346	11.2	379	12.2	987	31.9	<0.001
Very good or good	3,661	33.3	1,688	15.3	1,738	15.8	3,912	35.6		5,068	33.7	1,940	12.9	2,138	14.2	5,890	39.2	

**Table 3:** Adjusted odd ratios (and 95% confidence intervals) in the multinomial logistic regression of smoking on peer‐related issues

	Girls' smoking									Boys' smoking								
	Smokes daily			Smokes occasionally			Has quit smoking			Smokes daily			Smokes occasionally			Has quit smoking		
	OR	95% CI	*p*	OR	95% CI	*p*	OR	95% CI	*p*	OR	95% CI	*p*	OR	95% CI	*p*	OR	95% CI	*p*
Difficulties with mates																		
Has difficulties	0.89	0.77−1.02	0.081	0.98	0.82−1.16	0.785	0.95	0.79–1.13	0.532	1.02	0.91−1.14	0.704	0.98	0.84−1.14	0.772	0.95	0.82−1.11	0.538
No difficulties with mates	1			1			1			1			1			1		
Having a close friend																		
Don't have friends	**0.44**	**0.36−0.53**	**<0.001**	**0.53**	**0.41−0.67**	**<0.001**	0.85	0.79−1.06	0.139	**0.45**	**0.40−0.52**	**<0.001**	**0.49**	**0.41−0.59**	**<0.001**	**0.63**	**0.54−0.75**	**<0.001**
Has a friend or friends	1			1			1			1			1			1		
Bullying status																		
Bullied bully	2.41	1.26−4.64	0.008	0.69	0.23−2.14	0.525	0.81	0.29−2.30	0.695	**2.11**	**1.62−2.75**	**<0.001**	1.60	1.09−2.30	0.017	1.22	0.83−1.80	0.319
Bully	2.00	1.27−3.14	0.003	1.92	1.12.3.29	0.018	1.57	0.88−2.78	0.126	**2.23**	**1.74−2.86**	**<0.001**	**1.89**	**1.36−2.62**	**<0.001**	1.63	1.17−2.27	0.004
Victim	0.98	0.76−1.25	0.852	0.72	0.50−1.02	0.066	0.99	0.72−1.36	0.934	1.03	0.84−1.27	0.778	0.85	0.62−1.16	0.311	0.96	0.72−1.28	0.786
Not bullied not bully	1			1			1			1			1			1		
Perceived health																		
Moderate or bad	**1.95**	**1.78−2.14**	**<0.001**	**1.28**	**1.13−1.44**	**<0.001**	1.06	0.94−1.21	0.326	**1.59**	**1.44−1.75**	**<0.001**	1.10	0.95−1.26	0.196	1.05	0.92−1.20	0.478
Good or very good	1			1			1			1			1			1		

Note

Adjusted for age, family type, mothers' education and fathers' education

Bold values indicate *p* > 0.001

The statistical analyses were conducted using IBM (Armonk, NY) SPSS statistics 23. Results from the multinomial regression analyses are presented as odds ratio (ORs) and their 95% confidence intervals. The level of statistical significance was set at *p* < 0.001 due to the large number of respondents.

## 4 RESULTS

### 4.1 Adolescent smoking prevalence

As shown in Table 1, 37% of girls were daily smokers and 36% of boys. Girls were also occasional smokers (15%) slightly more often than boys (13%). Almost equal numbers, 15% of girls and 14% of boys, said they had quit smoking. Every third girl (33%) and almost four out of every 10 boys (38%) reported being non‐smokers.

### 4.2 Bivariate associations between peer relations and adolescent smoking compared with non‐smokers

In Table 2, we present the cross‐tabulation and chi‐square tests of smoking behaviour according to the peer relations variables and self‐rated health variable. Difficulties with schoolmates were not associated with smoking in either girls or boys. Instead, having a close friend or friends was significantly associated with smoking in both genders. Isolates, that is adolescents without a friend in whom they could confide, were less frequently daily smokers and were more often non‐smokers.

Being a victim of bullying at school was significantly associated with smoking in boys but not in girls. Participation of bullying behaviour was associated with smoking behaviour in both genders. Bullying status was significantly associated with smoking in both genders, and bullies were more frequently daily smokers than their classmates who did not participate in bullying behaviour.

Self‐rated health was significantly associated with smoking behaviour in girls and boys. Adolescents who assessed their health as moderate or poor were more often daily smokers than their counterparts who rated their health as follows: fairly good or good.

### 4.3 Multivariate associations between the peer relations variables, self‐perceived health and smoking behaviour

Multivariate associations between the peer relations and adolescent smoking and perceived health, for girls and boys respectively, are presented as ORs, and estimates are adjusted for the age of the respondent, parent's education level and family type (Table 3). Even after adjustment for these socio‐demographic characteristics and having a close friend and bullying status, difficulties with mates were not associated with smoking behaviour in either gender. However, having a close friend or friends added to the odds of girl's daily smoking and occasional smoking and to boys' daily smoking, occasional smoking and former smoking.

Adjusted model of bullying behaviour was not associated with smoking in girls. Boys who bullied others and were bullying victims themselves (bullied bully) were significantly more often daily smokers. In addition, bullies who were not themselves bullied (bully–not bullied) were significantly more often daily and occasional smokers. Being a victim (bullied–not bully) was not associated with smoking.

Adjusted (Table 3) multivariate regression analysis revealed that those girls who rated their health as moderate or bad smoked daily and occasionally significantly more often than their non‐smoking classmates. Boys who assessed their health as moderate or poor were significantly more often daily smokers.

## 5 DISCUSSION

In this nationally representative sample of Finnish 14‐ to 20‐year‐old vocational school students, peer relations and self‐rated health were associated with smoking behaviour in both girls and boys studying in upper secondary vocational schools. After controlling for the respondents age, family type and parents education level, this study identified: (a) having a friend or friends but not necessarily a class mate increased the odds for girls' and boys' smoking either daily or occasionally as well as being an ex‐smoker; (b) difficulties in relations with classmates were not associated with smoking behaviour; (c) being a bully increased the odds for smoking daily and occasionally and being a bully‐victim increased the odds for daily smoking only in boys; and d) poorer self‐rated health was associated with smoking behaviour in both girls and boys.

A literature review reported that isolates, that is adolescents without close friend (s), were more likely to smoke than their counterparts with a better peer network structure (Seo & Huang, 2012). This differs from the findings presented here. In this study, having at least one close friend was associated with higher odds of daily smoking in girls and boys and additionally in girls with occasional smoking. In this study, the smoking status of a friend was not investigated but according to previous research friends who smoke, peer influence and crowd affiliation (lähteet) increase adolescent smoking and also might explain some of the high rates of smoking adolescents in upper vocational schools. However, some social psychology theories might explain why adolescents smoke with friends and why smoking is more common in surroundings where smoking is more ubiquitous. These theories hypothesize that people can be categorized as belonging to groups, and they make social comparison with members of their own group—a process called social identification (Tajfel, 1981). Smokers identify themselves as part of the “smokers group,” and in their social comparisons, they make a distinction between us and them; that is between smokers and non‐smokers. It is possible that the fear of losing social status, being excluded from a group of people with similar values and attitudes will eventually become a part of their self‐identity. This may prevent established smokers from quitting smoking even though their awareness of the disadvantages of smoking is obvious. Furthermore, educational campaigns which hope reduce smoking by highlighting the fact that it is an abnormal habit might increase the gap between the groups of smokers and non‐smokers; in this case, these campaigns evidently cause more harm than benefit.

A recent study of Danish vocational school students indicated that smoking plays a significant role in social interactions and making new relationships across educational programmes, in other words, for example students from the painting programme or hairdressing can meet students from the carpentering and plumbing programmes. In that study, the vocational school context enhanced the likelihood of smoking; students took up smoking as a way of establishing social relationships with peers, and non‐smoking could lead to exclusion from relationships forged around an ashtray (Ingholt et al., 2015). Surprisingly, we did not find any association between difficulties with schoolmates and smoking behaviour. This may indicate that smoking is seen a way to fit in and conduct social relations. (Osgood, Feinberg, Wallace, & Moody, 2014; Suh, Shi, & Brashears, 2017) Instead, it is possible that difficulties with schoolmates can lead to withdrawal from the group of classmates.

In this study after controlling for respondents age, family type and parents' education bullying was related only with smoking in boys; both being a bully and being a bully who has also been a bullying victim were associated with smoking. An unanticipated finding was that among girls either participating in bullying behaviour or being a victim was not associated with smoking. In fact, bullying was not very widespread in vocational schools. On the other hand, it is possible that students underreported bullying. Another explanation for these results is that different aspects of peer relations other than bullying may increase the odds of smoking behaviour in upper vocational school. Smoking may be more prevalent with popular students and bullying is not considered as desirable behaviour, and being a bully is not a successful way to seek the positive attention of popular students.

We found that smokers evaluated their health as poorer than their non‐smoking classmates. Our research did not cover the age started experimenting with smoking but earlier studies have reported daily and occasional smokers to experience more health complaints and to have a lower quality of life than quitters, respectively (Dube et al., 2013; Hansen et al., 2015; Tian et al., 2016; Wang et al., 2012). Previously, it has been found that students report poorer subjective health if they had initiated smoking before the age of 14 than later starters. Established smokers are broadly aware of the addictive nature of cigarettes and the health consequences of cigarette smoking causes, even more so than their non‐smoking counterparts. However, smokers underestimate the addictive potency of nicotine and assure themselves that they will be able to quit before their health becomes compromised. (Twigg & Byrne, 2015) Furthermore, a longitudinal study has shown that adolescents with more than six smoking friends report increasing perceptions of benefits of smoking over time (Morrell, Song, & Halpern‐Felsher, 2010). Students in vocational school invariably rate peer relations as being more important than their health; however, addiction may come as surprise.

### 5.1 Limitations

Although the size, demographic coverage and long‐term stability of the SHP are impressive, there are limitations that give rise to caution. First, the cross‐sectional design prevents us from determining causality. Longitudinal data would have allowed us to examine temporal relationships between variables and the onset and progression of students' smoking behaviour. A second limitation is the usage of self‐report data; we cannot ignore the possibility of under‐ or over‐reporting of problematic behaviours (Brener, Billy, & Grady, 2003). Nevertheless, little data were available on peer relations and smoking behaviour outside of the self‐report paradigm. However, self‐reports have been shown to be reliable when conducted under optimized measurement conditions, ensuring anonymity and when using a variety of questions (Brener et al., 2003; Caraballo, Giovino, & Pechacek, 2004). Our approach could have underestimated the prevalence of bullying, because bullying has been associated with truancy (Havik et al., 2015) and students who are often absent from school may not have been included in the survey. Additionally, total of 12.5% or the respondents left the question of whether they had been bullied or not, unanswered. Last, despite the many advantages of secondary data, researchers are limited to the data collected during the original data collection. The primary data set was insufficient concerning missing data estimation; therefore, missing data could not be measured reliably. However, a clear strength of the primary data collection was that it was collected from every vocational institute in Finland. In this secondary analysis, the numbers of values which had to be excluded were low (between 0.3%–2.3%).

## 6 CONCLUSION

This study provides convincing evidence of the associations of peer relations and self‐reported health and smoking behaviour in the vocational school setting in a nationally representative sample. We found that friendships and bullying were robustly associated with an increased probability of smoking behaviour. Furthermore, daily smoking girls and boys and occasional smoking girls rated their health more often as only moderate or bad compared with their non‐smoking classmates. This new evidence highlights the importance of taking account of peer relations as well as the norms and expectations that peer relations might create for smoking. Therefore, schools should: (a) consider executing smoking‐related education and programmes for quitting using peer groups; and (b) enhancing social relationships and increasing the opportunities for social activities in the school and work together with students towards creating a healthy study environment. Further studies should consider using longitudinal data and investigate the relationship between peer relations and smoking behaviour in the vocational school setting with qualitative data.

## CONFLICTS OF INTEREST

No conflict of interest has been declared by the authors.
